# Evaluation of strategies for evidence-driven genome annotation using long-read RNA-seq

**DOI:** 10.1101/gr.279864.124

**Published:** 2025-04

**Authors:** Alejandro Paniagua, Cristina Agustín-García, Francisco J. Pardo-Palacios, Thomas Brown, Maite De Maria, Nancy D. Denslow, Camila J. Mazzoni, Ana Conesa

**Affiliations:** 1 Institute for Integrative Systems Biology, Spanish National Research Council, Paterna 46980, Spain;; 2 Department of Computer Science, Universitat de València, Valencia 46100, Spain;; 3 Department of Evolutionary Genetics, Leibniz Institute for Zoo and Wildlife Research, 10315 Berlin, Germany;; 4 Berlin Center for Genomics in Biodiversity Research, 14195 Berlin, Germany;; 5 Department of Physiological Sciences, Center for Environmental and Human Toxicology, University of Florida, Gainesville, Florida 32611, USA

## Abstract

While the production of a draft genome has become more accessible due to long-read sequencing, the annotation of these new genomes has not been developed at the same pace. Long-read RNA sequencing offers a promising solution for enhancing gene annotation. In this study, we explore how sequencing platforms, Oxford Nanopore R9.4.1 chemistry or Pacific Biosciences (PacBio) Sequel II CCS, and data processing methods influence evidence-driven genome annotation using long reads. Incorporating PacBio transcripts into our annotation pipeline significantly outperformed traditional methods, such as ab initio predictions and short-read-based annotations. We applied this strategy to a nonmodel species, the Florida manatee, and compared our results to existing short-read-based annotation. At the loci level, both annotations were highly concordant, with 90% agreement. However, at the transcript level, the agreement was only 35%. We identified 4906 novel loci, represented by 5707 isoforms, with 64% of these isoforms matching known sequences in other mammalian species. Overall, our findings underscore the importance of using high-quality curated transcript models in combination with ab initio methods for effective genome annotation.

The development of long-read, single-molecule sequencing technologies, such as Pacific Biosciences and Oxford Nanopore Technologies with the ability to produce ultra–long reads, has radically transformed our capacity for obtaining high-quality, even telomere-to-telomere, genome drafts and has boosted the establishment of several large-scale initiatives to sequence the genomes of all species on Earth. Projects such as Darwin Tree of Life (Blaxter 2022), the Vertebrate Genome Project (Rhie et al. 2021), and the Earth BioGenome Project (EBP) (Lewin et al. 2018) have undertaken the ambitious goal of sequencing the planetary biodiversity by establishing new protocols and pipelines for DNA extraction, sequencing, and genome assembly suitable to any nonmodel or poorly characterized species.

As the catalog of newly sequenced genomes increases, there is a concomitant necessity for genome annotation, which has not evolved at the same speed and still represents a bottleneck in our efforts to define the gene pools of living organisms (Yandell and Ence 2012). Some of the reasons behind this are the difficulty in annotating large fragmented “draft” genomes, the occurrence of annotation mistakes due to errors and contaminations in draft assemblies (Salzberg 2019), the lack of reference data for many nonmodel species, and the intrinsic uncertainty in the discovery of functional coding and noncoding elements in newly assembled genomes (Yandell and Ence 2012).

Currently, genome annotation approaches can be broadly divided into three main types: evidence-based methods, ab initio, and evidence-driven gene predictions (Yandell and Ence 2012). Evidence-based annotation or evidence alignment methods use experimental data, such as RNA-seq, protein sequences, or expressed sequence tags (ESTs) from the organism of interest or related species, which are mapped to the genome to identify genes (Yandell and Ence 2012; Jung et al. 2020). For this approach, RNA-seq has been shown to overperform other forms of evidence, since this type of data is able to capture splice sites, exon, and alternative spliced exon boundaries (Yandell and Ence 2012). However, evidence-based methods have several downsides, one of them being the amount of data available and tissues from which samples can be obtained (Liang et al. 2009; Scalzitti et al. 2020), which is oftentimes limited for nonmodel organisms, resulting in incomplete annotations. Another drawback of these methods is the propagation of incorrect annotations when using information from related species (Scalzitti et al. 2020). Recently, long-read RNA sequencing (lrRNA-seq) has started to be used for this purpose (Peng et al. 2022; Zhang et al. 2022). lrRNA-seq has the potential to capture full-length transcripts and reveal the complexity of the transcriptomes. However, lrRNA-seq data also contain sequencing and library preparation errors and benchmarking studies have demonstrated limitations of these data to deliver accurate transcript models (Pardo-Palacios et al. 2024b).

Ab initio approaches use mathematical models, like support vector machines (SVMs) or hidden Markov models (HMMs), to predict genes from the genomic sequence (Yandell and Ence 2012; Scalzitti et al. 2020). In this strategy, the models combine signal sensors (e.g., splice sites or polyadenylation signals) and content sensors (e.g., nucleotide composition or length of exons and introns) to make the predictions (Huang et al. 2016; Scalzitti et al. 2020). The main advantage of this approach is that no experimental evidence is needed (although it can be used, see next section) to detect genes and their genic structures (Yandell and Ence 2012), allowing the discovery of unidentified genes (Scalzitti et al. 2020). However, ab initio prediction programs tend to be error-prone, detecting noncoding nucleotides in exons (Scalzitti et al. 2020) and have low accuracy when predicting gene structures (Yandell and Ence 2012). Another limitation is that the majority of predictor tools tend to identify genetic coding sequences (CDSs) rather than untranslated regions (UTRs) or alternative isoforms, rendering incomplete annotations. In addition, ab initio methods need a trained model, which ideally should be organism-specific (Yandell and Ence 2012), and is not always available (Yandell and Ence 2012; Scalzitti et al. 2020).

Evidence-driven genome annotation is a powerful alternative for improving the detection of genes in genomes. In this case, experimental evidence such as gene or protein expression is used as support during the gene predictions by the ab initio programs, in order to increase their precision and overcome the sensitivity limitations of the evidence-alone methods. Several tools, such as AUGUSTUS (Stanke and Waack 2003; Stanke et al. 2006) and SNAP (Korf 2004), and popular annotation pipelines, like BRAKER (Stanke et al. 2008; Hoff et al. 2019) and MAKER (Campbell et al. 2014) have successfully implemented this strategy. Moreover, although the majority of genomes have been and are still being annotated using short-read RNA-seq as evidence, lrRNA-seq is increasingly being generated to serve this purpose. Potentially, long reads used as transcriptional data for evidence-driven approaches could overcome some of the limitations of the short reads to faithfully inform complete gene structures. However, no extensive studies have been carried out to evaluate the best strategy for using lrRNA-seq in the evidence-driven approach, leading to a disconnect between the latest sequencing technologies and the genome annotation pipelines (Cook et al. 2019).

In this work, we investigate various alternatives for evidence-driven genome annotation using lrRNA-seq data. Specifically, we evaluate different sequencing technologies (Pacific Biosciences [PacBio] Sequel II and Nanopore R9.41. chemistry) and read preprocessing levels (from raw reads to reconstructed transcripts and gene models). For benchmarking purposes, we used the well-annotated human genome and long-read cDNA reads of the human cell line WTC11 generated by the Long-read RNA-seq Genome Annotation Assessment Project (LRGASP) (Pardo-Palacios et al. 2024b). We exemplify the utility of the best-performing approach on the annotation of the nonmodel species, *Trichechus manatus latirostris* (Florida manatee).

## Results

**Figure 1.**
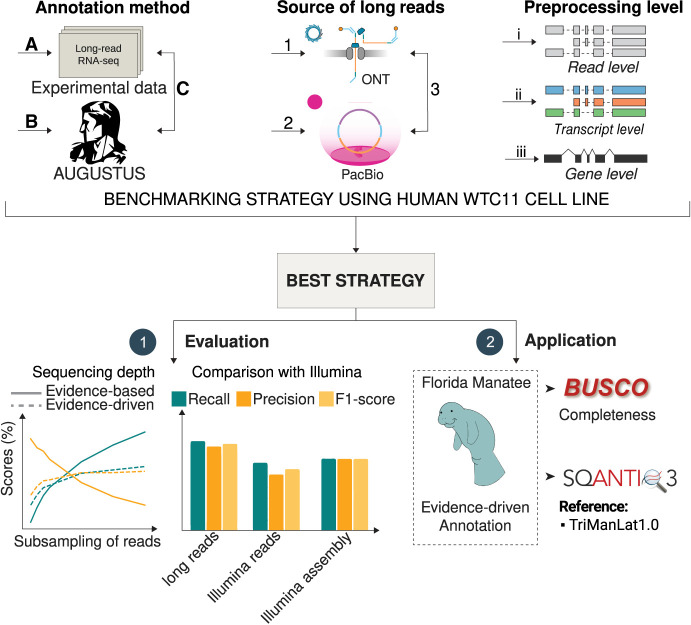
Overview of the evidence-driven, ab initio, and evidence-based annotation using long-read technologies. We compared three genome annotation strategies, evidence-based annotation using lrRNA-seq data (*A*), ab initio gene prediction using AUGUSTUS (*B*), and evidence-driven annotation combining both (*C*). We tested Nanopore (1) and PacBio (2) data independently and in combination (3) with processing levels ranging from raw reads (i) to transcripts (ii), and collapsing to the gene level (iii). For the best strategy defined in our benchmark, we evaluated the effect of the sequencing depth and compared to evidence-driven annotation using Illumina short-read data. Finally, we applied our strategy to a nonmodel species, the Florida manatee, applying BUSCO and SQANTI3 to evaluate the completeness of our annotation and comparing to the available Florida manatee annotation.

Evidence-driven genome annotation adds extrinsic evidence to ab initio algorithms to improve gene prediction. This approach requires careful consideration of both the modeling and prediction stages at which the evidence is incorporated, in addition to the type of supporting data used. We evaluated the utilization of lrRNA-seq data at both stages, the type of sequencing technology employed, and the level of data preprocessing. To provide a sound benchmarking scenario, cDNA long-read data from the human WTC11 cell line was used and results were compared to the GENCODE human annotation (Supplemental Fig. S1A,B). After determining the best procedure for training and prediction, we assessed the volume of sequencing data required to achieve optimal outcomes and compared this strategy to evidence-driven gene predictions supported by Illumina short reads (Supplemental Fig. S1C). Lastly, we applied the long-read evidence-driven approach to the annotation of the Florida manatee genome, recently sequenced by the LRGASP consortium (Pardo-Palacios et al. 2024b), using blood and brain lrRNA-seq data as experimental evidence sources. We then compared the long-read supported genome annotation to the existing NCBI *T. manatus latirostris* Annotation Release 102 annotation obtained using short reads (Fig. 1).

### Long-read sequencing data for curated gene set generation and HMM training

We first evaluated the utilization of long reads as evidence for model training using the human WTC11 cell line data from LRGASP. Training the HMM model requires the use of a set of high-quality, nonredundant gene models, which in this case should be obtained from the long reads. Due to the potential noisiness of raw lrRNA-seq data, we envisioned three levels of data preprocessing and assessed their suitability as reliable training sets. The first level represents a minimal read preprocessing scenario aiming at removing read redundancy by collapsing them by their junction pattern into *unique junction chain* (*UJC*) sequences, without further read correction or filtering. The second preprocessing level used the transcript models reconstructed with a suitable lrRNA-seq analysis pipeline (Iso-Seq3 for PacBio data, and FLAIR for Nanopore and PacBio + Nanopore data sets) coupled to basic filtering to provide a set of reliable error-corrected transcripts that included alternative isoforms. The third preprocessing level collapsed transcripts belonging to the same gene in one transcript model per gene to reduce sequence redundancy. See Supplemental Table S1 for a detailed description of the number of elements at each step. The characteristics of the data resulting from these three preprocessing levels were inspected by running SQANTI3 and BUSCO analyses.

**Figure 2.**
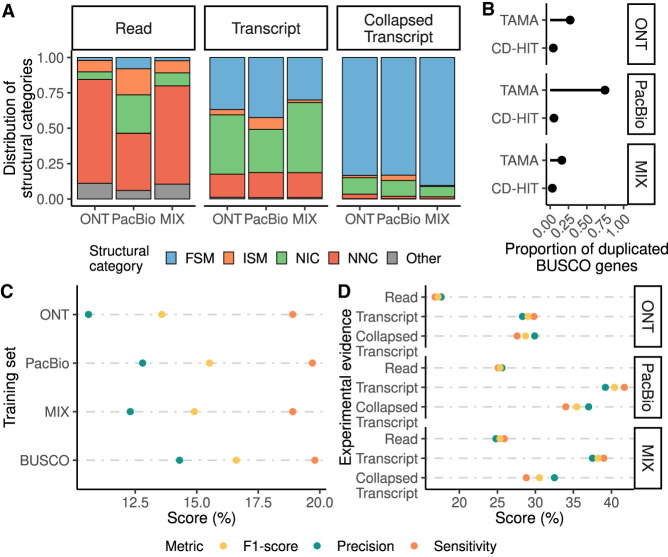
Assessment of the incorporation of long-read data to evidence-driven annotation. (*A*) Distribution of SQANTI3 categories at the read unique splice-junctions combination, transcript, and collapsed transcript level for PacBio data processed using Iso-Seq3 and ONT and ONT + PacBio data processed using FLAIR. (FSM) Full-Splice-Match, (ISM) Incomplete-Splice-Match, (NIC) Novel-in-Catalog, (NNC) Novel-Not-in-Catalog. (*B*) Redundancy of the generated transcriptome and the final collapsed transcripts set based on the proportion of duplicated BUSCO genes. (*C*) Evaluation of gene predictions by the three models trained with long-read data and the model trained using BUSCO genes. (*D*) Selection of the different long-read-based extrinsic evidence sources used for the evidence-driven gene predictions. Reads of PacBio, ONT, and a MIX of both (Read); transcript models of PacBio, ONT, and a MIX of both (Transcript) and collapsed transcripts identified in those transcriptomes (Collapsed Transcript) were used.

Using SQANTI3, we found that most UJC had at least one splice-site that was not present in the reference, i.e., were Novel-Not-in-Catalog (NNC) transcripts, whether we considered Nanopore, PacBio reads, or the combination of both (Fig. 2A). In contrast, the transcript reconstruction pipelines generated transcriptomes with a significant reduction in the proportion of NNC at the transcript level (Fig. 2A). One key aspect of our pipeline was the removal of monoexons, as between 62.5% and 82.1% of the obtained monoexons did not match any known transcript and were overly present in the two transcriptomes containing Oxford Nanopore Technologies (ONT) data (Supplemental Fig. S2). Other filtering criteria included in our pipeline were the presence of coverage of splice-junctions by short reads and the number of long reads associated with each transcript. Moreover, we kept only those transcripts with at least one BLAST hit, a query coverage over 85% and *E*-value <1 × 10^−50^. This, combined with previous filtering steps, reduced the proportion of NNC transcripts to below 10% for the PacBio transcriptome generated using Iso-Seq3 and the two FLAIR transcriptomes using either ONT data or a combination of ONT and PacBio reads (MIX). At this preprocessing level, most transcripts were either contained in the reference (Full-Splice-Match, FSM), or displayed a novel combination of annotated donor and acceptor sites (Novel-In-Catalog, NIC), indicating an improvement in the reliability of the transcript models.

The third preprocessing level aimed at reducing gene redundancy, which was still high, especially for the PacBio transcriptome. Nonredundancy is important to avoid overfitting during training (Hoff and Stanke 2019). To reduce the redundancy, we collapsed transcripts using TAMA and then applied a second clustering step at the protein level using Cluster Database at High Identity with Tolerance (CD-HIT). We evaluated the efficiency of this last preprocessing step using the BUSCO data set eutheria_odb10 including 11,366 genes. For the Iso-Seq transcriptome, the fraction of duplicated BUSCO genes was reduced from an initial value of 0.75 at the transcript level to 0.05 after clustering using CD-HIT. We obtained similar results for the ONT and MIX transcriptomes, with a final proportion of duplicated BUSCO genes of 0.04 and 0.03, respectively (Fig. 2B), suggesting a successful collapse of transcripts into gene models without compromising the discovery of true BUSCOs (Supplemental Fig. S3).

Next, we used the three sets of collapsed nonredundant transcripts, derived from the PacBio, ONT, and MIX long reads, to train the ab initio model and assessed their performance. For completeness, we included a fourth training set consisting of the BUSCO genes identified in the human genome, as this is a common strategy for ab initio gene prediction (Simão et al. 2015). Generally, to evaluate the performance of the gene prediction models, the gene set is divided into training and test sets. The training set is used to train the model, which is then evaluated by predicting genes in the test set and comparing predictions to the true gene annotations. However, it has been shown that this evaluation method overestimates the performance of the models (Guigó et al. 2000). To address this problem, rather than using a long-read-derived test set, we reannotated Chromosome 19 of the human genome. The predictions obtained with the trained models were compared to the 1420 protein-coding genes in the reference annotation of Chromosome 19. Performance was evaluated as sensitivity, precision, and F1-score at the nucleotide, exon, and gene levels. We considered predicted genes as true positives (TPs) only if they matched the CDS of a gene in the reference annotation from start to end positions, with all splice sites correctly annotated. To identify the best set of parameters for each type of data, we tested different values for the flanking region used to learn noncoding region patterns and the number of genes included in the training set. We found that, in general, shorter flanking regions (∼1000 bp) and a training set size of 4000–5000 genes provided the best performance results, beyond which no further improvement was observed. Additionally, some data-type-specific behaviors were noted (Supplemental Fig. S4). This pattern was observed at the nucleotide, exon, and gene levels and was especially notable for the BUSCO training set.

We then evaluated the performance of the best model obtained with each data type. The F1-score at the gene level ranged from 0.13 to 0.16 (Fig. 2C), with models obtained by BUSCO and PacBio data scoring the highest. Sensitivity was very similar between the four models, while the precision for the BUSCO training set was slightly better (Fig. 2C). Next, we characterized the exon number and length of the predictions. We denoted as TP genes those with all their junctions and ends matching a gene in the reference, partial true positive (PTP), those with partial overlap with a reference gene, false genes (FGs), those gene predictions that did not overlap with any gene in the reference, and missed genes, the genes that were completely missed by the gene prediction algorithm. The challenges associated with ab initio gene prediction tools are well-documented, as these tools tend to overestimate the presence of genes (Wang et al. 2003; Drăgan et al. 2016; Scalzitti et al. 2020). We found that the number and length of exons in these four types of predicted genes were consistent among the different training sets. TP genes had generally fewer and longer exons than other groups (Supplemental Fig. S5A,B), revealing that these gene structures are easy to predict. False negative (FN) genes, on the contrary, while displaying a similar number of exons, had shorter exons (Supplemental Fig. S5A,B). This finding agrees with research by Scalzitti et al. (2020) which demonstrated that AUGUSTUS accuracy decreases for genes with short exons. Finally, FGs typically had fewer but longer exons compared to all predictions (Supplemental Fig. S5A,B), with BUSCO training resulting in the lowest (635) and ONT in the highest (1082) number of FGs. Based on these results we concluded that, at least for our experimental settings, long-read-based (LRB) transcript models did not provide an advantage over employing BUSCO genes for AUGUSTUS ab initio gene prediction and selected this last approach for our subsequent analyses.

### PacBio transcript models achieve higher performance when used as experimental evidence in the gene prediction step

Once established as the best approach for model training, we evaluated the utilization of lrRNA-seq data at the gene prediction step in the evidence-driven strategy implemented by AUGUSTUS. We reannotated Chromosome 19 of the human genome HMM model trained with BUSCO genes and provided as experimental evidence each of our three types of long-read preprocessed data: (1) full-length nonconcatemer (FLNC) PacBio reads and raw ONT long reads independently and in combination; (2) filtered protein-coding transcripts obtained with transcript reconstruction algorithms, and (3) collapsed transcripts derived from those transcriptomes. Note that AUGUSTUS developers recommend using PacBio long reads with minimum processing for evidence-driven annotations (Hoff and Stanke 2019).

We found that the utilization of reads as experimental evidence, particularly from the ONT platform, resulted in the lowest precision and sensitivity (Fig. 2D). We hypothesize that this outcome was due to the presence of reads with unannotated splice sites or aligning to noncoding regions, which lead to FG predictions, supporting the notion that preprocessing of raw reads improves evidence-driven annotation. Employing transcript models as external evidence provided superior performance than the nonredundant, collapsed transcripts set. In particular, the best performance was achieved using the transcriptome inferred from PacBio data as experimental evidence. This transcriptome recovered 959 protein-coding genes encoded in Chromosome 19, whereas the collapsed version of this transcriptome recovered only 587 genes. The combined PacBio and ONT transcriptome achieved a slightly inferior F1-score compared with the results obtained with the PacBio transcriptome, whereas the ONT transcriptome resulted in the lowest F1-score (Fig. 2D).

These results show that the genome annotation substantially improves when long reads are processed into transcripts models or collapsed transcripts and provided as experimental evidence to ab initio algorithms for prediction, improving both sensitivity and precision.

### Evidence-driven lrRNA-seq genome annotation reaches an optimal trade-off between sensitivity and precision with increasing sequencing depth

Once we established that processing long reads into transcript models was the best choice for leveraging lrRNA-seq data in genome annotation, we asked to which extent the amount of sequencing throughput impacts annotation. We sampled various proportions of the total PacBio reads and conducted both evidence-based and evidence-driven annotation procedures, using the annotation of human Chromosome 19 as ground truth. We evaluated sensitivity and precision as in previous sections but additionally studied the proportion of missed loci. A locus was considered missed if no annotation features overlapped it. This metric demonstrates how the presence of imperfect but potentially meaningful predictions changes with sequencing depth in the two annotation approaches.

**Figure 3.**
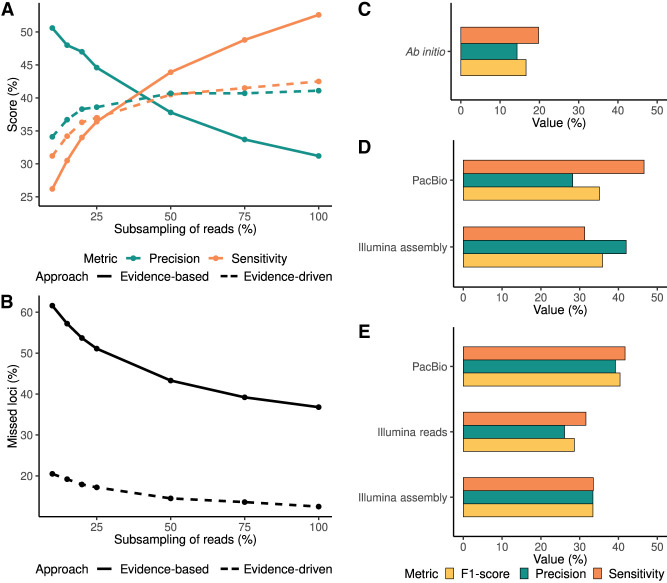
Performance analysis of gene prediction as a function of the number of reads. Sensitivity, precision (*A*), and number of missed loci (*B*) were obtained with different sample sizes of WTC11 cell line PacBio FLNC reads using evidence-based and evidence-driven approaches. Performance of the different genome annotation approaches with Illumina short-read and PacBio long-read technologies at the gene level. (*C*) Ab initio predictions. (*D*) Evidence-based models using PacBio and Illumina-assembled transcriptomes. (*E*) Evidence-driven approach with PacBio, Illumina reads or Illumina-assembled transcriptomes as the source of evidence for the prediction step.

While the evidence-based model achieved higher sensitivity, the precision dropped drastically when more data were incorporated (Fig. 3A). On the contrary, the evidence-driven approach resulted in both metrics reaching a plateau when approximately half of the data were included in the predictions, representing 3,501,946 FLNC reads. Despite the sensitivity being improved when more reads were used, this increase was not as pronounced as the values obtained with the evidence-based approach. However, the precision obtained with the evidence-driven annotation method increased as more data were added at the prediction step, compared with the precision values of the evidence-based approach. In this sense, the evidence-driven strategy reached a trade-off between sensitivity and precision, limiting the incorporation of low-quality predictions and redundancy in the annotation. In addition, the evidence-driven approach was able to capture more loci even when small amounts of data were provided as extrinsic evidence (Fig. 3B). This contrasted with the evidence-based strategy, where despite increasing recall as more reads were used, the number of loci captured was still lower than the evidence-driven annotation method, especially at the lowest sequencing depth. We concluded that the evidence-driven approach overcomes one of the main limitations of evidence-based methods, which is the amount of available data, while providing a better balance between precision and recall.

These results collectively highlight the superiority of evidence-driven annotation approaches over purely experimental methods in achieving a good balance between sensitivity and precision when working with limited lrRNA-seq data.

### The evidence-driven approach outperforms the rest of genome annotation approaches when long-read technologies are used

We finally asked if evidence-driven genome annotation using long reads outperforms traditional approaches such as ab initio methods or annotation based on short reads. For this, we annotated the human Chromosome 19 using Illumina data in both evidence-driven and evidence-based strategies. Moreover, we evaluated the utilization of either Illumina raw reads, as recommended by AUGUSTUS developers, or a short-read assembled transcriptome as the source of experimental evidence. Performance was compared to the PacBio transcriptome evidence-based and evidence-driven annotation and with the AUGUSTUS ab initio approach.

As expected, the lowest precision and sensitivity were obtained with the ab initio method (Fig. 3C), possibly due to the identification of fragmented and FGs, as frequently described (Scalzitti et al. 2020). Notably, when considering evidence-based approaches, employing an Illumina-assembled transcriptome resulted in higher precision than using PacBio transcripts, while the sensitivity achieved with the long-read transcript models was higher compared with Illumina. The F1-score of both evidence-based approaches was very similar, 35.1% and 35.9% for the PacBio and Illumina transcriptomes, respectively (Fig. 3D). In the case of the evidence-driven methods, the lowest precision and sensitivity values were obtained when raw Illumina reads were used as the source of evidence. This again underscores the limitations of raw reads as evidence source for evidence-driven genome annotation as shown for the long reads. Despite the improved F1-score when Illumina transcript models were provided for evidence-driven annotation, the best performance of this strategy was achieved by employing the PacBio transcriptome. Furthermore, the combination of the evidence-driven method and long-read technologies outperformed the rest of the approaches achieving a balance between sensitivity and precision leading to the highest F1-score (Fig. 3E). We also attempted to incorporate short-read data to retain transcript models with coverage of all their splice-junctions; however, the combination of short- and long-read data did not yield improved results (Supplemental Table S2).

Altogether, these results show that the combination of long-read-derived transcript models with the evidence-driven annotation method provides superior results than other strategies, establishing new guidelines for genome annotation

### Evidence-driven annotation of the Florida manatee genome

**Figure 4.**
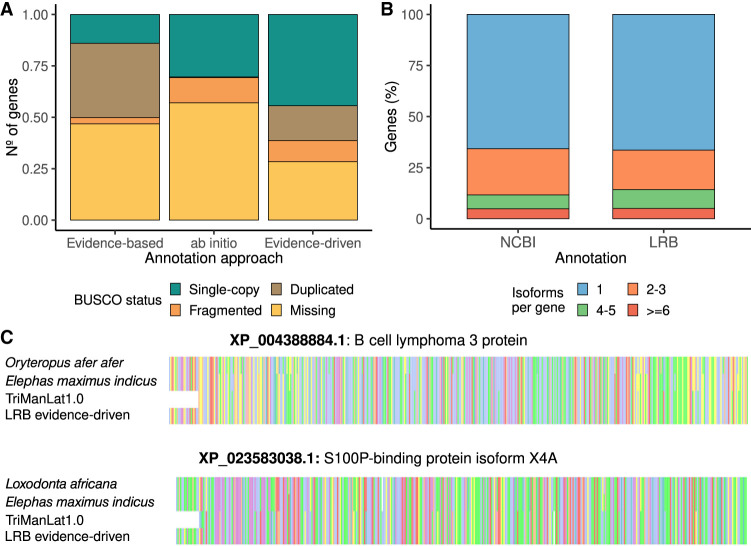
LRB annotation of the Florida manatee genome. (*A*) Assessing the BUSCO completeness of the Florida manatee's genome annotation with the different approaches. (*B*) Number of isoforms per gene in the NCBI annotation (*left*) and the LRB annotation (*right*). (*C*) Multiple sequence alignments of proteins of two manatee-related species, the sequence identified in the new annotation, and the manatee sequence available at NCBI. At the *top*, B cell lymphoma 3 protein is shown. At the *bottom*, S100P-binding protein isoform X4 is shown.

Given that the PacBio transcriptome evidence-driven strategy achieved the highest sensitivity and precision for gene prediction in the human genome, we used this approach for the annotation of the Florida manatee genome draft obtained by the LRGASP consortium (Pardo-Palacios et al. 2024b). We compared the results with other annotation methods by analyzing BUSCO completeness. This analysis measures the proportion of genes that are complete (C), either as single-copy (S), or duplicated (D), as well as fragmented (F) and missing (M) genes, out of the total 11,366 genes in the Eutheria_odb10 data set. The available PacBio lrRNA-seq data set consisted of 5,434,382 FLNC blood and 2,709,782 FLNC brain reads. Using the Iso-Seq pipeline, we generated 461,040 transcript models. SQANTI3 filtering was applied to remove transcripts with noncanonical splice-junctions, intrapriming artifacts, and RT-switching (Cocquet et al. 2006) while retaining only multiexon transcripts with CDS longer than 300 nt. Although a high-quality reference gene annotation was unavailable, we showed in the well-annotated WTC11 data set that this feature-based filtering effectively reduced the proportion of NNC and intergenic transcripts. Following this stringent filtering, we identified 54,491 high-confidence transcript models. This final BUSCO completeness was C:50.1%[S:14.0%, D:36.1%], F:3.1%, M:46.8%, n:11,366 (Fig. 4A).

Following our benchmarking guidelines, the AUGUSTUS HMM model was trained with the BUSCO genes identified in the LRGASP Florida manatee genome. This model predicted 15,735 genes on the manatee genome, with a final BUSCO completeness of C:30.7%[S:30.4%, D:0.3%], F:12.3%, M:57.0%, n:11,366. After adding the PacBio transcriptome during the prediction step for an evidence-driven strategy, 35,662 transcripts and 20,782 genes were predicted representing BUSCO completeness of C:61.3%[S:44.4%, D:16.9%], F:10.3%, M:28.4%, n:11,366 (Fig. 4A). Compared to the evidence-based method, the evidence-driven approach achieved a higher BUSCO completeness with almost 20,000 fewer transcripts. These results are in agreement with the results obtained during the human benchmark, where the evidence-driven method controlled the number of missed loci obtained while balancing precision and sensitivity.

To further complete the annotation, we incorporated the BUSCO genes identified in the manatee genome with the PacBio transcriptome and used these two sources of evidence in combination during the gene prediction step. This strategy resulted in a BUSCO completeness of C:87.9%[S:56.4%, D:31.5%], F:4.4%, M:7.7%, n:11,366 and contained a total of 21,082 genes and 39,977 transcripts. This transcriptome was used for downstream analysis.

### Comparison of the long-read-based manatee genome annotation with the NCBI annotation

We compared our LRB annotation to the available NCBI *T. manatus latirostris* Annotation Release 102, obtained using short reads and the NCBI Eukaryotic Genome Annotation Pipeline, to evaluate any gain or loss of information. We first determined the number of isoforms per gene in each of the annotations. Although the NCBI and LRB annotation had a similar proportion of genes that only encode one isoform (65.73% and 66.42%, respectively), we noticed that the number of genes coding multiple isoforms was slightly different. The public annotation contained a higher number of genes with two or three isoforms (22.62%) compared to the LRB one (19.26%). In contrast, the proportion of predicted genes at the LRB annotation with four or more isoforms was 14.31%; while in the case of the NCBI annotation, this percentage was 11.64% (Fig. 4B).

To further understand these differences, we then used the SQANTI3 framework to classify the LRB transcript models when compared against the NCBI annotation as reference annotation, and the NCBI transcripts compared against the LRB models as reference. In this comparison, identical annotations are identified as FSM in both directions, and differences are revealed as other SQANTI3 transcript categories (Supplemental Fig. S6A). The reciprocal comparisons returned 35.11% of transcripts as FSM when assessing NCBI annotation against the LRB reference, while 24.11% were FSM when LRB models were compared to the NCBI annotation. In addition, 85.7% of the transcripts in the LRB transcriptome and 90.2% of the transcripts in the NCBI annotation could be assigned to loci found in both annotations. In these shared loci, transcripts classified as Incomplete-Splice-Match represent new isoforms with alternative TSS or TTS, while NIC and NNC indicate transcripts with different splicing patterns (Supplemental Fig. S6A). These results revealed that, despite an overall good agreement between the two annotations in the identification of loci, transcript models differed substantially. Specifically, only 35% of transcripts in the NCBI annotation and 25% in the LRB annotation were classified as FSM when reciprocally compared. This suggests that the source and/or strategy for incorporating experimental evidence, different in each case, strongly impacts annotation results.

Next, we focused on the isoforms assigned to novel loci in each annotation. Using cuffcompare, we identified 4906 novel loci exclusively present in the LRB annotation and 2225 loci unique to the NCBI annotation. To better characterize these loci, we evaluated the support for the isoforms found in these loci using known sequences from other mammalian species. Of the 5707 isoforms of the LRB annotation associated with novel loci, 3671 had at least one BLAST hit against the UniProt Mammalia curated protein database with an *E*-value <1 × 10^−3^. Of the total 2036 transcripts without a BLAST hit, 693 (34.04%) were supported by experimental evidence, while for the transcripts with a BLAST hit, 2481 (67.58%) were supported (Supplemental Fig. S6B). In comparison, for the NCBI annotation, 3137 out of 3212 isoforms had at least one BLAST hit. These results indicate the capacity of long-read methods to support the annotation of novel genes, while also highlighting the known risk of false discoveries associated with ab initio methods (Scalzitti et al. 2020).

Next, we characterized events of gene fragmentation, where genes are fragmented in one annotation compared to the other. An example is shown in Supplemental Figure S6C. In this case, we found that the public annotation had 1610 isoforms overlapping multiple genes of the new annotation, while the LRB annotation only has 496 putative fusion isoforms, suggesting a higher level of fragmentation in the LRB annotation. We hypothesized that this potential fragmentation may result from the ab initio predictions in the absence of experimental evidence. However, 1117 out of the total 1610 NCBI fusion isoforms overlapped multiple experimentally supported LRB genes. These experimentally supported fragmented LRB genes exhibited a similar median length and number of exons as the other supported LRB isoforms (Supplemental Fig. S6D,E). Since the distribution of lengths and number of exons of these LRB fragmented genes was similar to the nonfragmented genes, we evaluated if the fragments originated from the same gene. We used BLAST to analyze fragmented genes of the LRB annotation overlapped by one NCBI isoform to determine if these supported genes were part of the same human gene. Of the 1087 NCBI fusion isoforms that overlapped supported LRB genes with at least one BLAST hit, 1041 had LRB genes matching the same BLAST hit. This result suggests that the LRB annotation included supported, but fragmented genes.

To understand if this fragmentation was already present in the experimental evidence provided for the predictions or caused during the gene prediction step, we used SQANTI3 to evaluate the experimental evidence of the fragmented supported genes of the LRB transcriptome. We detected 100 isoforms from the experimental data that overlapped multiple genes of the LRB annotation, corresponding to 54 different loci. Of these 54 loci, 17 lacked experimental evidence to support any of the fragmented genes, while in the other 37 cases, at least one of the fragmented genes was supported by the experimental data (Supplemental Fig. S7A,B). These results suggest that while gene fragmentation can occur during gene prediction, the primary cause is the presence of fragmented experimental evidence. Since we did not observe evidence of gene fragmentation when benchmarking our evidence-based approach, we asked if differences in the quality of the manatee lrRNA-seq data with respect to the human WTC11 PacBio reads could explain the different behavior. We found a substantial shift toward shorter reads in the manatee blood and brain transcriptome data when compared to the WTC11 cell line, possibly reflecting a poorer library preparation quality for the manatee field sample (Supplemental Fig. S8A). Accordingly, we found that fragmented genes corresponded to transcript models with CDSs lengths above 1.5 kb, which were depleted in the manatee data set (Supplemental Fig. S8B). We concluded that biases in transcript capture by long-read methods may compromise the capacity for providing supporting evidence in evidence-driven annotation methods.

Finally, we evaluated if LRB annotation predicted proteins could potentially improve the protein sequences of the public annotation. We selected 2166 isoforms from the NCBI annotation that specifically matched the splice-junctions of an isoform in the LRB annotation, either fully (FSM) or partially (ISM), and exhibited a truncation of at least 50 bp at one end. Among the 1597 matched LRB isoforms, 1002 (66.4%) had a BLAST hit in the UniProt curated mammalian database, with differences of fewer than three amino acids at the protein ends. We further aligned the sequences of two of these truncated proteins, their matching LRB proteins, and their orthologs in two closely related manatee species. Figure 4C shows the alignment data for B cell lymphoma 3 protein and the S100P-binding protein isoform X4. In both cases, the protein sequence in the NCBI annotation lacked an N-terminal fragment present in the LRB version and the two manatee-related species. These results confirm the potential of the LRB annotation to improve the current gene annotations.

## Discussion

Long-read sequencing technologies, concerted with global efforts to sequence all Earth's organisms, herald a new era of possibilities and requirements for genome annotation. Traditionally, genome annotation has relied on ab initio algorithms alone or combined with short-read sequencing and proteomics data to improve gene predictions. However, with the increased availability and throughput of lrRNA-seq, there is a notable shift toward using transcriptome data generated from these platforms to assist genome annotations in nonmodel species, as exemplified in several recent studies involving the tea plant (Xia et al. 2019) and the ant *Harpegnathos saltator* (Shields et al. 2021).

Long-read sequencing offers the advantage of producing full-length transcripts, potentially providing a more complete representation of gene models than is obtained with short reads. However, challenges such as sequencing errors and library artifacts can compromise the reliability of long-read data. Initiatives like the LRGASP have demonstrated that de novo transcript reconstruction using solely long-read data still faces significant hurdles (Pardo-Palacios et al. 2024b).

In response to these challenges, we hypothesized that long reads could be optimally used in evidence-driven strategies, where experimental evidence is integrated with ab initio genome prediction algorithms to enhance their efficacy. Aware of the limitations of long-read transcriptome sequencing, our study aimed to assess how lrRNA-seq data can be best used to support genome annotation efforts. Our results indicate that processing lrRNA-seq data into transcript models followed by SQANTI3 curation, rather than using raw reads, and providing this evidence information at the prediction step is the most effective strategy, possibly because the reconstructed transcript models represent more accurate transcript structures than the raw reads. Additionally, we observed that PacBio Sequel II sequencing data yielded better results than ONT v9.4.1 chemistry data or a combination of both. This result is in agreement with the conclusions of the LRGASP project (Pardo-Palacios et al. 2024b), that revealed that the longer read distribution and sequence accuracy of the long reads were more important for accurate transcript model prediction than the number of reads. Our results suggest that this is also true when using long-read transcript models for evidence-driven annotation. Moreover, our comparative analysis of results with human and manatee lrRNA-seq data suggests that narrow read length distributions can compromise the accurate support of longer gene models, resulting in gene fragmentation. These results stress the importance of ensuring high-quality full-length transcript models to support genome annotation pipelines.

While our study provides important insights regarding the utilization of long-read sequencing technologies in genome annotation, we acknowledge several limitations in our work. First, we provided AUGUSTUS with relatively simple lrRNA-seq data sets (one cell line in human and two tissues for the manatee) to evaluate genome prediction accuracy. Genome annotation efforts often use multiple tissue types to capture the broadest range of expressed genes, and our study did not extensively assess this aspect. We acknowledge the need for future studies to explore the impact of tissue diversity on annotation accuracy. However, obtaining diverse tissue samples can be challenging for certain nonmodel species, as this was the case for the manatee, where access to multiple tissue samples was extremely difficult. Our results suggest that only moderate amounts of long-read data are necessary to enhance ab initio prediction accuracy and sensitivity, and that the combination of the ab initio with the available lrRNA-seq evidence is an effective way to overcome the problems associated with the limited data.

Additionally, in this work, we only evaluated FLAIR and Iso-Seq tools for transcript model construction, as these are widely used tools, and both can operate without reference annotations. However, other methods exist for long-read transcript reconstruction (Bushmanova et al. 2019; Sahlin and Medvedev 2020; Wyman et al. 2020; Tian et al. 2021; Chen et al. 2023; Lienhard et al. 2023; Nip et al. 2023; Prjibelski et al. 2023; Volden et al. 2023). As the LRGASP assessment indicated that de novo reconstruction of transcript models from lrRNA-seq data still poses challenges, a follow-up study should evaluate the performance of other algorithms for evidence-driven annotation.

The LRGASP consortium demonstrated the impact of the tools and sequencing technologies in the identification of different transcripts. In our case, the differences observed between the LRB manatee annotation and the NCBI annotation are likely influenced by not only the distinct types of RNA-seq data used but also by the differences in the annotation pipelines employed.

Finally, while we do not claim that the genome annotation strategy outlined in this study represents the best possible pipeline for lrRNA-seq-based genome annotation, the major takeaway of this work, namely, the utilization of high-quality curated transcript models rather than raw long reads in conjunction with ab initio methods as most effective approach for genome annotation offers a valuable perspective for ongoing global efforts to develop quality annotation pipelines using long-read sequencing to annotate the planet's biodiversity.

## Methods

### Sample acquisition, nucleic acid extraction, and sequencing

Total RNA was extracted from the brain tissue of an adult West Indian manatee (*T. manatus*), sourced from a captive individual at Tierpark Berlin. The extracted RNA displayed a RIN of 7.8. A PacBio Iso-Seq library was prepared from this RNA sample and sequenced using a single SMRT cell.

### Obtaining transcripts from RNA sequencing data

#### Assembly of WTC11 short-read data

Quality control and adapter trimming of the reads was performed using fastp v0.23.2 (Chen et al. 2018). This involved the detection and removal of adaptors from the reads, ensuring data integrity and accuracy. Following preprocessing, reads were aligned to the reference genome using STAR v2.7.10a (Dobin et al. 2013). To enhance splice junction quantification, the alignment process used the ‐‐twopassMode option (Veeneman et al. 2016).

To generate a short-read assembled transcriptome, the reads were aligned using TopHat2 (Kim et al. 2013) with the options ‐‐no-discordant, ‐‐no-mixed, and -r 400, to accommodate a fragment length of 600 bp with an end length of 100 bp. Subsequently, the mapped reads of each sample were individually assembled using StringTie2.2.1 (Pertea et al. 2015) with default parameters. Finally, a nonredundant set of transcripts was generated by executing StringTie in merge mode, utilizing default parameters. Only stranded transcripts were kept.

### Reconstruction of WTC11 and Florida manatee long-read transcriptomes

The Iso-Seq pipeline was used to generate transcript models from PacBio long-read data. This pipeline includes preceding subreads into circular consensus sequencing (CCS) reads (ccs v6.0.0). FLNC reads were obtained from the CCS reads after removing the sequencing primers (lima with ‐‐isoseq and ‐‐peek-guess options) and the poly(A) tails (isoseq refine with ‐‐require-polya option). After that, the FLNC reads of the samples were clustered (isoseq cluster with ‐‐verbose ‐‐use-qvs options). The high-quality clustered reads were then mapped to the genome using minimap2 v2.17 (Li 2018). Finally, the mapped reads were collapsed into unique isoforms (isoseq collapse with ‐‐do-not-collapse-extra-5exons option). Only those transcript models supported by at least two reads were kept.

To obtain the WTC11 ONT transcriptome and the ONT + PacBio (MIX) transcriptome we used FLAIR v1.5.1 (Tang et al. 2020). Long reads were aligned to the human genome using the FLAIR align module with default options. Splice-junctions were corrected using the FLAIR correct module, incorporating the splice-junctions generated by STAR during short-read alignment. Splice-junctions with at least three supporting short reads were kept and used to correct the long-read splice-junctions. The corrected long reads were then collapsed into transcript models using the FLAIR collapse module with the options -s 2 –stringent –check_splice –filternosubset to reduce the number of redundant isoforms per gene.

### Transcriptome filtering

SQANTI3 v4.2 (Pardo-Palacios et al. 2024a) was used to filter reconstructed transcriptomes and eliminate artifacts. We incorporated Illumina short reads as orthogonal data with the -c parameter. Filtering consisted of the removal of monoexons, transcripts with nonsense mediated decay signals, transcripts with noncanonical splice-junctions, and transcripts with evidence of intraprimming. We retain transcripts with coding potential identified by GeneMarkS-T (Tang et al. 2015) and an open reading frame (ORF) of at least 300 nt. For the long-read defined transcriptomes, only transcripts with at least two associated long reads were retained. BUSCO v5.4.7 (Manni et al. 2021) was run in transcriptome mode using the Eutheria_odb10 data set, including 11,366 genes, on these transcriptomes. The BUSCO completeness results were expressed as C:89.0%[S:85.8%, D:3.2%], F:6.9%, M:4.1%, n:11,366, where C represents the percentage of complete genes found in the input data, S indicates the percentage of these genes found as single copies, D represents the percentage of duplicated genes, F stands for fragmented genes, M refers to missing genes, and n denotes the total number of genes in the BUSCO data set used.

### Benchmarking of gene prediction methods on WTC11

The software AUGUSTUS v3.1 (Hoff and Stanke 2019) was used for ab initio, evidence-based, and evidence-driven gene prediction strategies. Evidence was obtained from the long-read or short-read data at three levels of processing: raw reads without preprocessing (raw level), reconstructed or assembled transcript models (transcript level), and collapsed transcripts into unique loci (gene level).

#### Obtaining training sets from long-read transcripts

To obtain a reliable and nonredundant set of long-read transcripts to use as training sets for AUGUSTUS, we further filtered the three transcriptomes based on the SQANTI3 output. Only transcript models with all their splice-junctions supported by short reads were included in the training set. For the ONT + PacBio transcriptome, we only kept the 25% most expressed transcripts. Moreover, protein sequences predicted by GeneMarkS-T in the filtered WTC11 transcript models were searched in the manually reviewed mammalian proteins available at UniProt (https://www.uniprot.org) using BLAST + v2.12.0 (Camacho et al. 2009) with an *E*-value cutoff of 1 × 10^−50^. Only proteins with at least one BLAST hit and a query coverage over 85% were kept for the training set.

To minimize the number of isoforms per gene, TAMA Collapse (Kuo et al. 2020) was applied to the three filtered WTC11 transcriptomes, utilizing options -x no_cap -m 100 -z 100. Subsequently, a second clustering, based on the sequence of the predicted proteins in the transcripts, was conducted using CD-HIT v4.8.1 (Fu et al. 2012). Sequences with an identity higher than 80% were clustered with the option -c 0.8. The gene coding the longest protein was kept as the representative of the cluster. Finally, BUSCO genes in Eutheria_odb10 data set were identified in the final training sets after clustering at the protein level.

#### AUGUSTUS HMM training and ab initio gene prediction with WTC11 data

The AUGUSTUS HMM was trained using nonredundant transcripts sourced from the Iso-Seq WTC11 PacBio transcriptome, the FLAIR WTC11 ONT transcriptome, or a combination of FLAIR PacBio and ONT WTC11 transcriptomes (MIX). Additionally, BUSCO genes in Eutheria_odb10 data set identified in the human genome were used for HMM training.

Various parameters were explored during training, including the number of genes in the training set and the length of the flanking region. Flanking region lengths of 1000, 2500, 5000, 7500, and 10,000 nt were tested. Training gene sets of sizes ranging from 200 to 5000 were generated from the three nonredundant training sets derived from the WTC11 long-read transcriptomes.

The AUGUSTUS script gff2gbSmallDNA.pl was employed to convert files from GTF to GenBank format, incorporating the appropriate flanking region. Evaluation of HMMs was conducted using Monte-Carlo cross-validation (Xu and Liang 2001). Each training gene set was randomly generated three times using different seeds, resulting in a total of 117 training sets.

#### Gene prediction with experimental evidence on the human genome

AUGUSTUS was run using the –softmasking=on option and different sources of experimental evidence to predict genes on Chromosome 19 of the human genome. The experimental evidence given to AUGUSTUS was: Illumina short reads, ONT and PacBio long reads individually and in combination, the Iso-Seq3 WTC11 PacBio transcriptome, the FLAIR WTC11 ONT transcriptome, the FLAIR PacBio and ONT WTC11 transcriptome, the Illumina transcriptome and the three sets of nonredundant collapsed transcripts generated from each of the three long-read transcriptomes.

For the four transcriptomes, only multiexon transcripts identified as protein-coding and with no features of bad quality, i.e., signals of nonsense mediated decay, signals of intrapriming, noncanonical splice-junctions, and a CDS shorter than 300 nt, were given to AUGUSTUS.

The default AUGUSTUS configuration file for long-read evidence was used as input for the raw long-read data. For the Illumina-assembled transcriptome and the long-read transcriptomes, only the CDSs identified by GeneMarkS-T were converted to AUGUSTUS hints. For the Illumina short reads aligned with STAR, the default AUGUSTUS configuration file for short-read evidence was used. Ultimately, these sets of predicted genes were compared to the reference human annotation of Chromosome 19 using Cuffcompare v2.2.1 (Trapnell et al. 2010).

#### WTC11 genome annotation evaluation

The performance of gene prediction strategies was evaluated by comparison to the annotation of the human Chromosome 19 using Cuffcompare v2.2.1 with the -e 0 -d 0 options. The reference annotation was constructed from the GENCODE annotation selecting the coding region of the transcript with the longest ORF for each gene on Chromosome 19.

A predicted gene was considered a TP when all exonic and genic nucleotides matched the corresponding features in the reference. Nucleotides, exons, or genes not matching any feature in the reference were considered false positives (FPs), while features in the reference not perfectly matched by gene predictions were categorized as FNs.

Cuffcompare provided the sensitivity (*Sn*) and precision (*Pr*) values at the nucleotide, exon, and gene levels. The F1-score was calculated as the harmonic mean of these two values.

$$Sn=\frac{\mathrm{TP}}{\mathrm{TP}+\mathrm{FN}};\;Pr=\frac{\mathrm{TP}}{\mathrm{TP}+\mathrm{FP}};\;F1\text{-}\mathrm{score}=2\times \frac{Sn\times Pr}{Sn+Pr}$$

#### Assessment of sequencing depth in evidence-based and evidence-driven annotation

To evaluate the effect of sequencing depth on genome annotation we performed evidence-driven and evidence-based annotation using an increasing proportion of the total WTC11 PacBio FLNC reads. Subsampling of the FLNC reads from the WTC11 cell line was conducted using SAMtools v1.16 (Danecek et al. 2021). The fraction of sampled reads was 0.1, 0.15, 0.2, 0.25, 0.5, 0.75, and the full data set. Following subsampling, the FLNC reads were processed using the Iso-Seq v4.0.0 pipeline. The same strategy described in the previous sections was used to filter the transcriptomes. CDSs identified using GeneMarkS-T were included in AUGUSTUS v3.1 as experimental evidence. Finally, the seven sets of predicted genes, alongside the experimental data, were compared to the reference human annotation of the Chr19 using Cuffcompare v2.2.1.

### Annotation of the Florida manatee genome using long reads

#### AUGUSTUS ab initio predictions on the manatee genome

The AUGUSTUS HMM was trained using BUSCO genes identified within the LRGASP Florida manatee genome (Pardo-Palacios et al. 2024b). The training gene set consisted of 5000 genes, with a flanking region length of 1000 nt. Files were converted from GTF to GenBank format with the appropriate flanking region using the AUGUSTUS script gff2gbSmallDNA.pl.

Subsequently, the trained model was used to predict genes on the manatee genome. For the ab initio predictions on the manatee genome, BUSCO was employed in protein mode, utilizing the Eutheria_odb10 data set.

#### Evidence-driven annotation of the manatee genome

AUGUSTUS v3.1 was executed with the ‐‐softmasking=on option, utilizing the CDSs identified by GeneMarkS-T within the filtered PacBio transcripts as extrinsic evidence. To predict several isoforms per gene, we included the ‐‐alternatives-from-evidence=on option.

Subsequently, the protein sequences of these predictions were obtained with the AUGUSTUS getAnnoFasta.pl script. For assessing the BUSCO completeness, BUSCO was run in protein mode, employing the Eutheria_odb10 and the protein sequences of the predicted genes obtained using the created transcriptome.

Finally, BUSCO genes identified in the genome were concatenated with the long-read evidence and provided to AUGUSTUS as hints for evidence-driven annotation. Protein sequences of the predictions were obtained to assess the BUSCO completeness of the annotation.

#### Comparison of the Florida manatee long-read-based genome annotation with the NCBI Trichechus manatus latirostris Annotation Release 102

To compare the NCBI short-read-based genome annotation of the Florida manatee with our LRB annotation, we first mapped the CDS sequences from NCBI to the LRGASP Florida manatee genome using minimap2 with the options -ax splice -uf –MD. After obtaining the primary sorted alignment using SAMtools v1.16, we generated the GFF file using spliced_bam2gff v1.2. Both annotations were compared using Cuffcompare v2.2.1 with options -e 0, -d 0, and -G. To validate the unique genes of each annotation, we extracted their protein sequences and used BLAST v2.13 to search these sequences on a custom-made database with representatives of all the mammalian proteins, using an *E*-value cutoff of 1 × 10^−3^.

To study the structural classification of isoforms in each annotation, SQANTI3 v5.2 was run using the liftOver of the NCBI annotation (only the protein-CDSs) as input and the complete LRB annotation as the reference annotation; and vice versa. We identified those isoforms classified by SQANTI3 as fusion and further characterized those cases in which NCBI isoforms overlapped multiple supported genes of the LRB annotation, i.e., the genes determined using both the long-read experimental data or BUSCO genes as support for AUGUSTUS during the prediction step. After filtering the supported predicted genes from the LRB annotation, we divided these genes into two groups based on whether they were part of a fusion isoform in the NCBI annotation or not. Then, the number of exons and length of these genes present in the LRB annotation were studied.

To determine if these supported LRB genes composing the NCBI fusion isoforms were fragments of the same protein, BLAST v2.13 was run using their protein sequences and utilizing a custom-made database with all the human proteins. The options used for running BLAST were an *E*-value cutoff of 1 × 10^−3^ and -num_alignments 10. The BLAST custom-made database was created with all the human-curated proteins available at UniProt. After that, a custom-made Python script was developed to test if the BLAST hits recovered were the same for the supported LRB genes overlapped by the same NCBI fusion isoform. To address whether the fragmentation was caused by the experimental evidence provided for the predictions or during the gene prediction step, SQANTI3 was run using the experimental data as input and the supported LRB genes overlapped by NCBI fusion isoforms as reference. In case of fragmentation during the gene prediction step, the experimental evidence would be classified as fusion, overlapping multiple genes.

#### Comparison with other species

To demonstrate the potential of our LRB annotation to improve the NCBI *T. manatus latirostris* Annotation Release 102, we selected transcripts with matching splice-junctions in both annotations but with truncated CDS in the NCBI annotation. We extracted the corresponding protein sequences and searched them using BLASTP v2.15 on the NCBI Reference Proteins (refseq_protein) database. The hits of two different species were selected based on these filters: lowest *E*-value and both highest query cover and percent of identity. The four sequences were aligned using Clustal Omega (Sievers and Higgins 2021) from the EMBL-EBI web page (Madeira et al. 2022).

### Data sets

Transcriptomics data used in this study were obtained from the LRGASP project and downloaded from the ENCODE database (https://www.encodeproject.org/), with the following accession: ENCSR673UKZ for the human WTC11 Illumina RNA-seq (three samples, 143,171,620 paired-end reads), ENCSR513JKI for the manatee Illumina RNA-seq (nine samples, 513,272,173 paired-end reads), ENCSR539ZXJ for WTC11 Nanopore raw reads (three samples, 30,664,338 reads), ENCSR507JOF for WTC11 PacBio raw subreads (three samples, 6,943,271 FLNC reads), and ENCSR272BQI (three Sequel II samples) and ENCSR583MLP (one Sequel I sample) for manatee peripheral blood mononuclear cells (5,434,382 FLNC reads). Additionally, this study generated a total of 2,709,782 FLNC reads from the brain sample of a single manatee individual.

The human reference genome used for lrRNA-seq and RNA-seq data processing and structural annotation was downloaded from the GENCODE database (https://www.gencodegenes.org/) (GRCh38.p13 release 38 reference primary assembly). The corresponding reference annotation was downloaded from GENCODE and filtered to contain only protein-coding genes. The LRGASP *T. manatus latirostris* genome, assembled using Nanopore and Illumina reads, was downloaded from the NCBI GenBank database (https://www.ncbi.nlm.nih.gov/) (GCA_030013775). The short-read-based TriManLat1.0 genome assembly (GCF_000243295.1) and the NCBI *T. manatus latirostris* Annotation Release 102 were downloaded from RefSeq (https://www.ncbi.nlm.nih.gov/refseq/).

## Data access

The transcriptomics data generated in this study have been submitted to the European Nucleotide Archive (ENA; https://www.ebi.ac.uk/ena/browser/) under accession number PRJEB76292. The custom scripts, final Florida manatee annotation, and AUGUSTUS hints generated in this study can be found on GitHub (https://github.com/alexpan00/lr_evidence_driven) and as Supplemental Code.

## Supplemental Material

Supplement 1

Supplement 2
