# Supplementary material for: Evaluation of strategies for evidence-driven genome annotation using long-read RNA-seq
Source: Genome Res. 2025 Apr;35(4):1053–64. doi: 10.1101/gr.279864.124 (PMC12047274; doi:10.1101/gr.279864.124)
Supplement: Supplement 1 [file Supplemental_Material.pdf]

Supplemental Material

Evaluation of strategies for evidence-driven genome annotation using long-read RNA-seq

Alejandro Paniagua[1,2*], Cristina Agustín-García[1,*], Francisco J. Pardo-Palacios[1], Thomas Brown[3,4], Maite De Maria[4,5], Nancy D. Denslow[4], Margaret E. Hunter[6], Camila J. Mazzoni[3,4], Ana Conesa[1]

[1]Institute for Integrative Systems Biology, Spanish National Research Council, Paterna, Spain

[2]Department of Computer Science, Universitat de València, Valencia, 46100, Spain

[3]Department of Evolutionary Genetics, Leibniz Institute for Zoo and Wildlife Research Berlin, Germany

[4] Berlin Center for Genomics in Biodiversity Research, Germany

[4] Department of Physiological Sciences and Center for Environmental and Human Toxicology, University of Florida, Gainesville, FL 32611, USA

[5] Cherokee Nation System Solutions, contractor to the U.S. Geological Survey, Wetland and Aquatic Research Center, Gainesville, FL USA

[6] U.S. Geological Survey, Wetland and Aquatic Research Center, Gainesville, FL USA

contact: ana.conesa@csic.es

* equally contributing

Table of Contents

Transcriptome filtering parameters

Supplemental Tables

Supplemental Table S1: Overview of the total number of reads and transcripts at different processing stages.

Supplemental Table S2: Impact of Short-Read Curation on Evidence-Driven WTC11 Transcriptome Annotation.

Supplemental Figures

Supplemental Figure S1: Overview of genome annotation strategies using the WTC11 dataset.

Supplemental Figure S2: Proportion of monoexon and multiexon isoforms.

Supplemental Figure S3. Evaluation of BUSCO completeness using the eutheria_odb10 dataset before and after transcriptome collapse via CD-HIT.

Supplemental Figure S4. Assessment of AUGUSTUS gene predictions as a function of the type of training set, the number of genes in the training set, and the length of the flanking region added to those genes.

Supplemental Figure S5. Characterization of *ab initio* gene predictions.

Supplemental Figure S6. Comparison of the Long-read-based (LRB) annotation of the Florida Manatee with the short-read-based NCBI annotation.

Supplemental Figure S7. Characterization of fragmented genes.

Supplemental Figure S8. Distribution of reads and CDSs lengths.

Transcriptome filtering parameters

General filtering was applied to the PacBio, ONT, and MIX WTC11 transcriptomes based on transcript features obtained from the output of SQANTI3. Transcripts were retained if they met the following conditions: exons > 1, coding == coding, predicted_NMD == FALSE, perc_A_downstream_TTS < 60, all_canonical == canonical, CDS_length > 300 and RTS_stage == FALSE. These filtered transcriptomes were subsequently used for evidence-based annotation.

For training the AUGUSTUS model, more stringent filtering was employed to ensure a highly reliable set of transcripts. In the case of the PacBio and ONT transcriptomes, only transcripts with a minimum coverage (min_cov) at splice-junctions exceeding the median value across the entire transcriptome were retained. For the MIX transcriptome, which contained the largest number of transcripts, the top 25% of transcripts with the highest min_cov and FL (full-length read support) were selected. Additionally, across all three transcriptomes, only transcripts encoding proteins with a BLAST hit in the curated mammalian proteome from UniProt, with at least 85% coverage, were included. Transcripts were collapsed, to minimize the number of isoforms per gene. TAMA Collapse was applied to the three filtered WTC11 transcriptomes, utilizing options -x no_cap -m 100 -z 100. Subsequently, a second clustering, based on the sequence of the predicted proteins in the transcripts, was conducted using CD-HIT v4.8.1 . Sequences with an identity higher than 80% were clustered with the option -c 0.8.

	PacBio	ONT	MIX
Reads	6,943,271	30,664,338	37,607,609
Unique combination of splice-junctions	543,000	2,062,103	2,451,431
Transcripts	145,945	133,115	170,935
Transcripts (evidence-driven annotation)	88,117	33,540	57,111
Transcripts (model training)	30,675	10,910	8,816
Collapsed transcripts (model training)	8,758	6,224	6,259

Supplemental Table S1. Overview of the total number of reads and transcripts at various stages of processing using PacBio, ONT, and their combination (MIX). The table includes the following processing levels: raw reads, unique splice-junction combinations, transcript models generated by Iso-Seq or FLAIR, filtered transcriptomes based on transcript features, transcripts further filtered by read coverage (short and long reads) and BLAST® hits, and the final collapsed transcriptome produced using TAMA and CD-HIT.

	Without short-reads		With short-reads	
	Sensitivity	Precision	Sensitivity	Precision
Nucleotide	82.8	91.9	81.9	91.9
Exon	74.2	85.6	72.7	85.3
Transcript	41.7	39.2	41.3	39

Supplemental Table S2. Sensitivity and precision metrics of the evidence-driven WTC11 transcriptome assessed at the nucleotide, exon, and transcript levels. PacBio transcripts were used as hints, both with and without filtering transcripts that have complete splice-junction coverage by short reads.

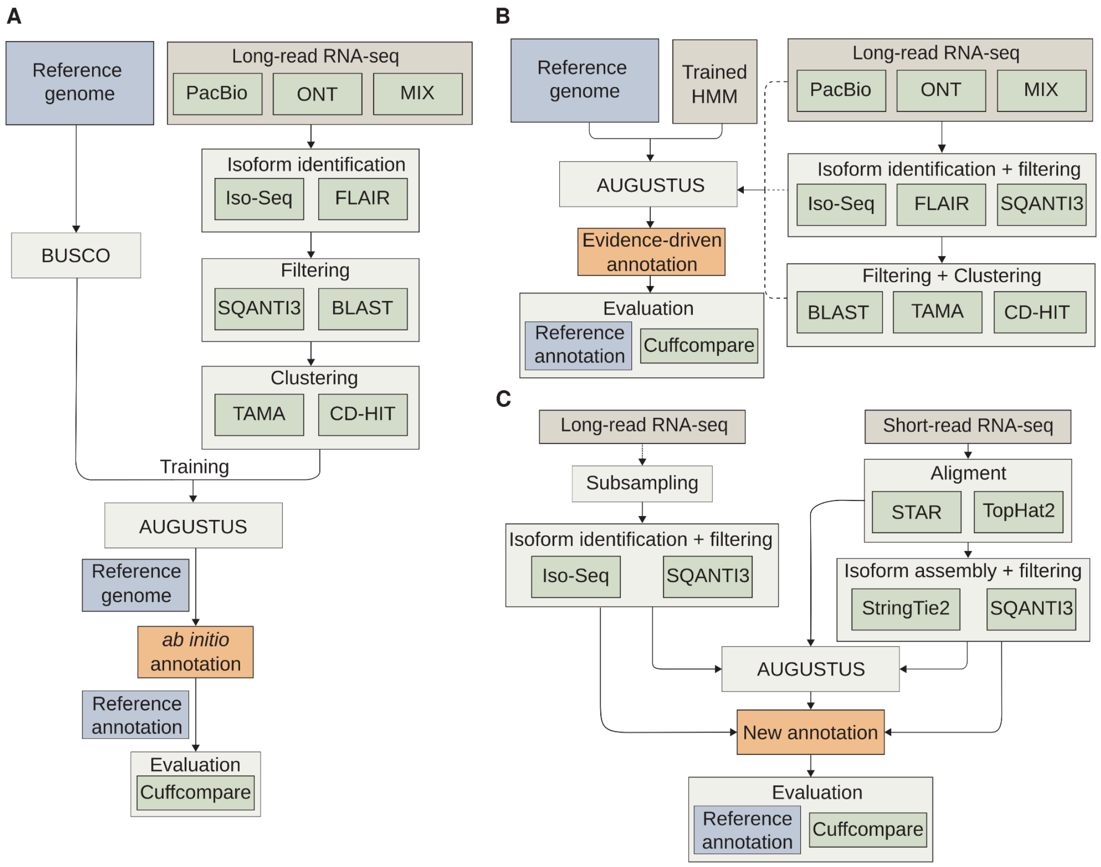

Supplemental Figure S1. Overview of genome annotation strategies using the WTC11 dataset. **(A)** Evaluation of the optimal training set for *ab initio* gene prediction. Long reads generated from PacBio and/or ONT platforms were processed into reliable, non-redundant transcripts. The initial step involved a transcript reconstruction pipeline using Iso-Seq for PacBio data, FLAIR for ONT data, and a combination of PacBio and ONT (MIX). SQANTI3 was applied to all three transcriptomes, and the SQANTI3 output, in combination with BLAST results, was used to filter the transcriptomes. To reduce redundancy, we employed TAMA and CD-HIT on the filtered transcriptomes. Subsets of transcripts with varying flanking regions were used to train the AUGUSTUS Hidden Markov Model (HMM). As an alternative approach, we also trained the AUGUSTUS HMM model using BUSCO genes identified in the human genome. The resulting models were then applied to predict genes on the reference genome, and the predictions were compared to the reference annotation using Cuffcompare. (B) Incorporating long-read data in the gene prediction step. After determining the optimal training strategy, we evaluated the impact of long-read data on gene prediction. We tested three levels of long-read

processing: raw reads, filtered isoforms generated by FLAIR or Iso-Seq, further filtered based on transcripts features provided by SQANTI3, and the same collapsed and filtered transcripts used for model training. The nine resulting annotations, generated using different levels of long-read data processing, were compared to the reference annotation using Cuffcompare. (C) Comparison of evidence-based and evidence-driven annotation using short and long reads. Long-read data were processed into transcript models and filtered based on transcript features. These transcripts were used either as input for AUGUSTUS in an evidence-driven annotation or were directly compared to the reference annotation. This process was repeated iteratively, with increasing proportions of total reads sampled. For short-read data, we assessed two strategies. First, the reads were aligned to the reference genome using STAR, and the resulting alignments were provided to AUGUSTUS for evidence-driven annotation. In the second strategy, we aligned the reads using TopHat2 and assembled the transcripts using StringTie2. These transcript models were filtered using the same criteria as for the long-read-derived transcripts. Finally, the assembled transcripts were either provided to AUGUSTUS for evidence-driven annotation or directly compared to the reference annotation.

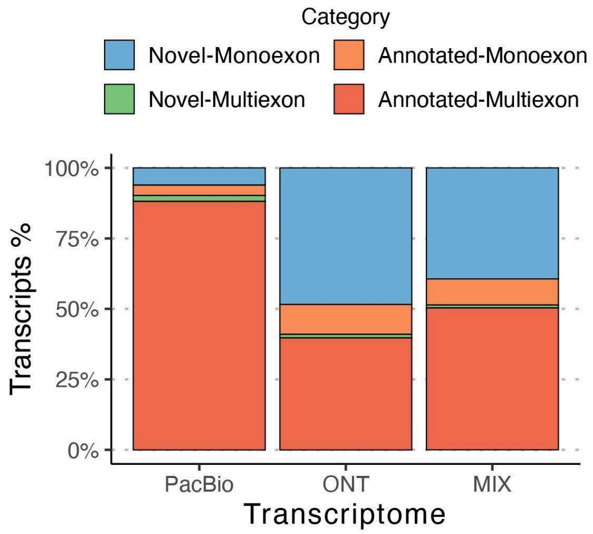

Supplemental Figure S2. Proportion of monoexon and multiexon isoforms. A large proportion of unannotated monoexons were discovered using FLAIR and ONT or MIX data. PacBio: PacBio transcriptome obtained with IsoSeq3 pipeline, ONT: Nanopore transcriptome obtained with FLAIR, MIX: Transcriptome combining PacBio and Nanopore reads obtained with FLAIR.

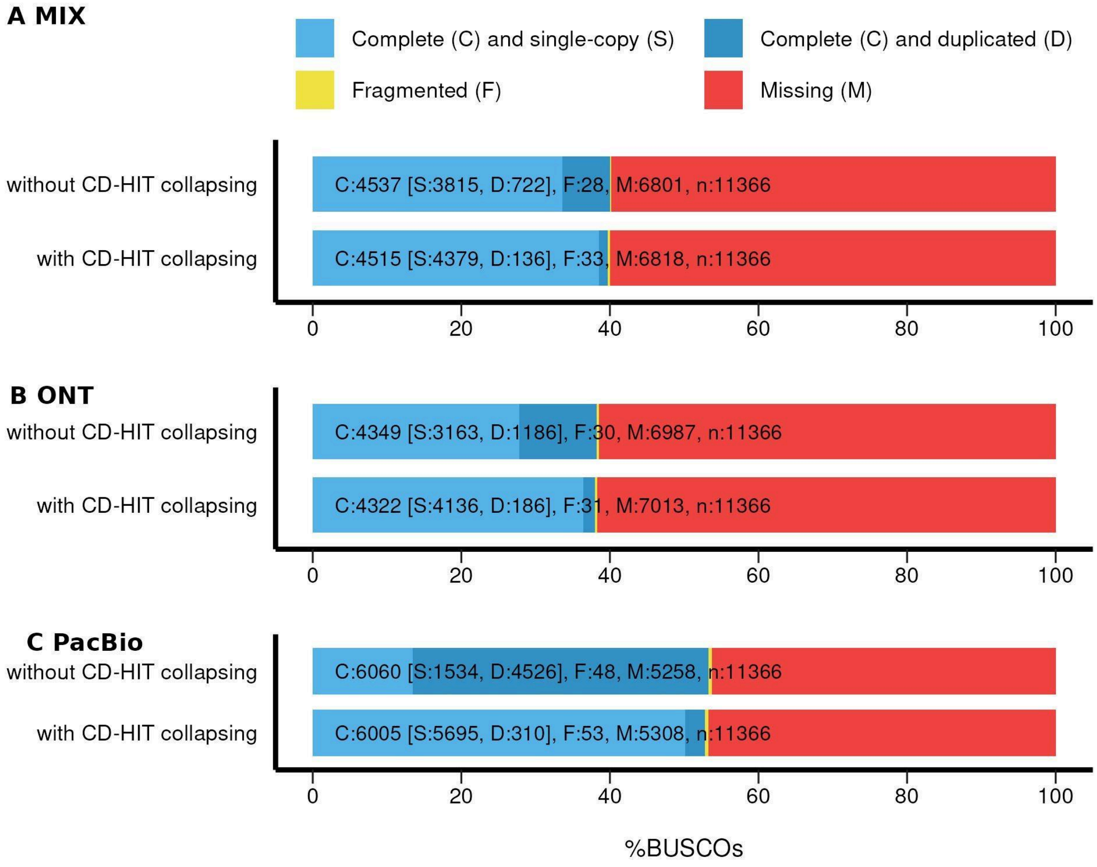

Supplemental Figure S3. Evaluation of BUSCO completeness using the eutheria_odb10 dataset before and after transcriptome collapse via CD-HIT. The proportion of duplicated BUSCO genes is significantly reduced, whereas the total number of identified genes remains largely unchanged for the 3 transcriptomes. PacBio: PacBio transcriptome obtained with IsoSeq3 pipeline, ONT: Nanopore transcriptome obtained with FLAIR, MIX: Transcriptome combining PacBio and Nanopore reads obtained with FLAIR.

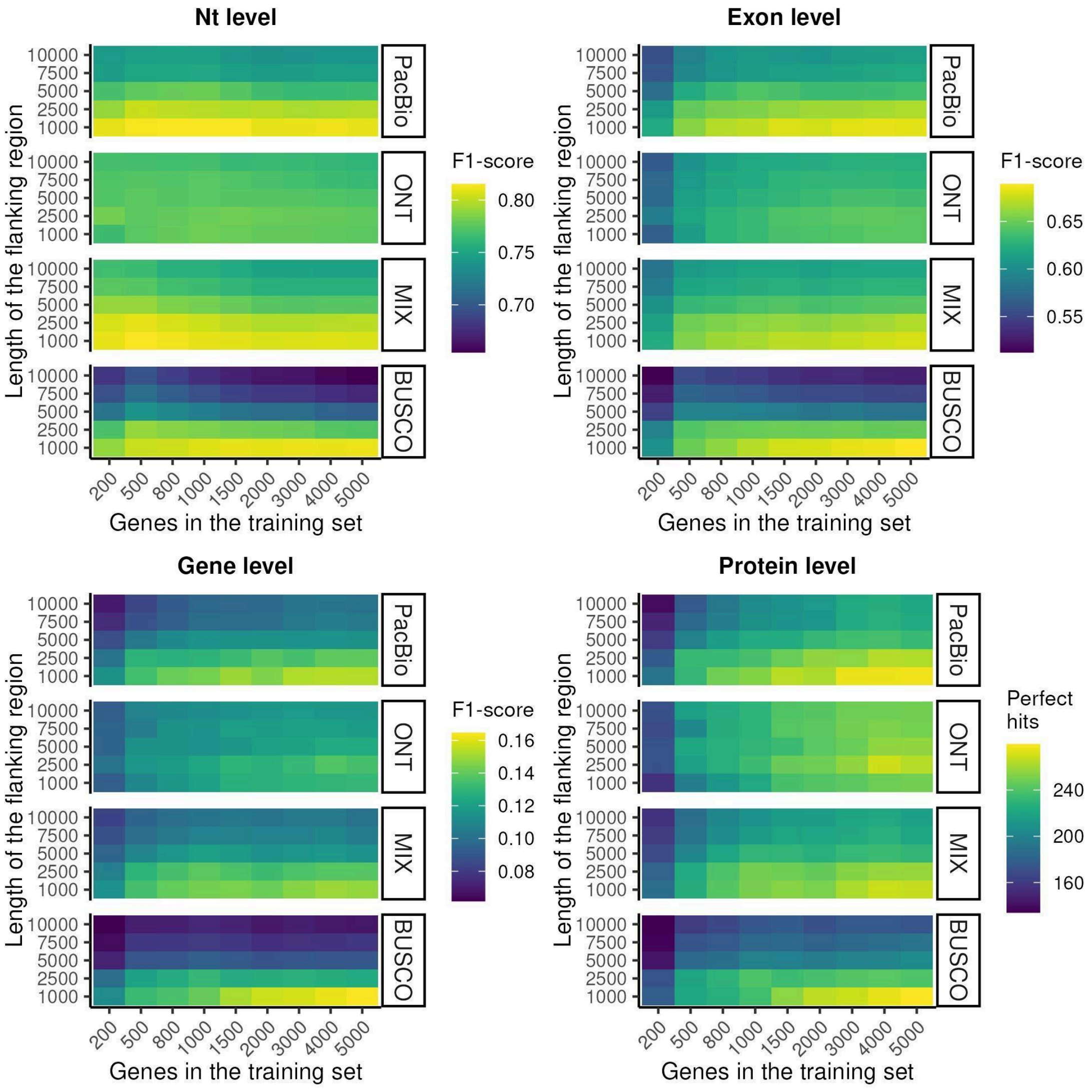

Supplemental Figure S4. Assessment of AUGUSTUS gene predictions as a function of the type of training set, the number of genes in the training set, and the length of the flanking region added to those genes. PacBio: PacBio transcriptome obtained with IsoSeq pipeline, ONT: Nanopore transcriptome obtained with FLAIR, MIX: Transcriptome combining PacBio and Nanopore reads obtained with FLAIR. BUSCO: Genes predicted by BUSCO analysis on the unannotated human genome.

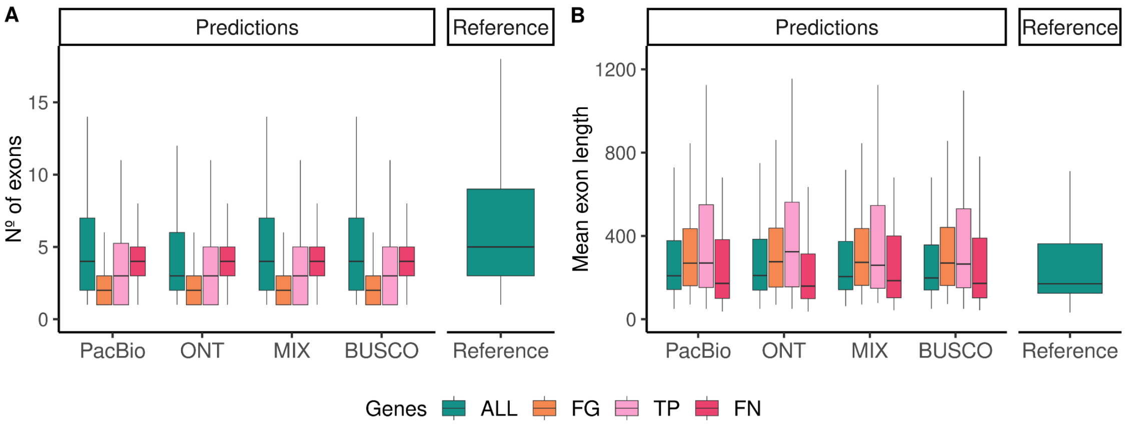

Supplemental Figure S5. Characterization of *ab initio* gene predictions. The number of exons (A) and their mean length (B) was compared between the predicted genes, the false negative (FN) genes and the reference annotation. The predicted false genes (FG) and true positive (TP) genes were separated from the rest of the predictions to evaluate their characteristics. PacBio: Model obtained with the PacBio/IsoSeq3 transcriptome, ONT: Model obtained with the Nanopore/FLAIR transcriptome, MIX: Model obtained with the transcriptome combining PacBio and Nanopore reads obtained with FLAIR. BUSCO: Model obtained with the genes predicted by BUSCO analysis on the unannotated human genome.

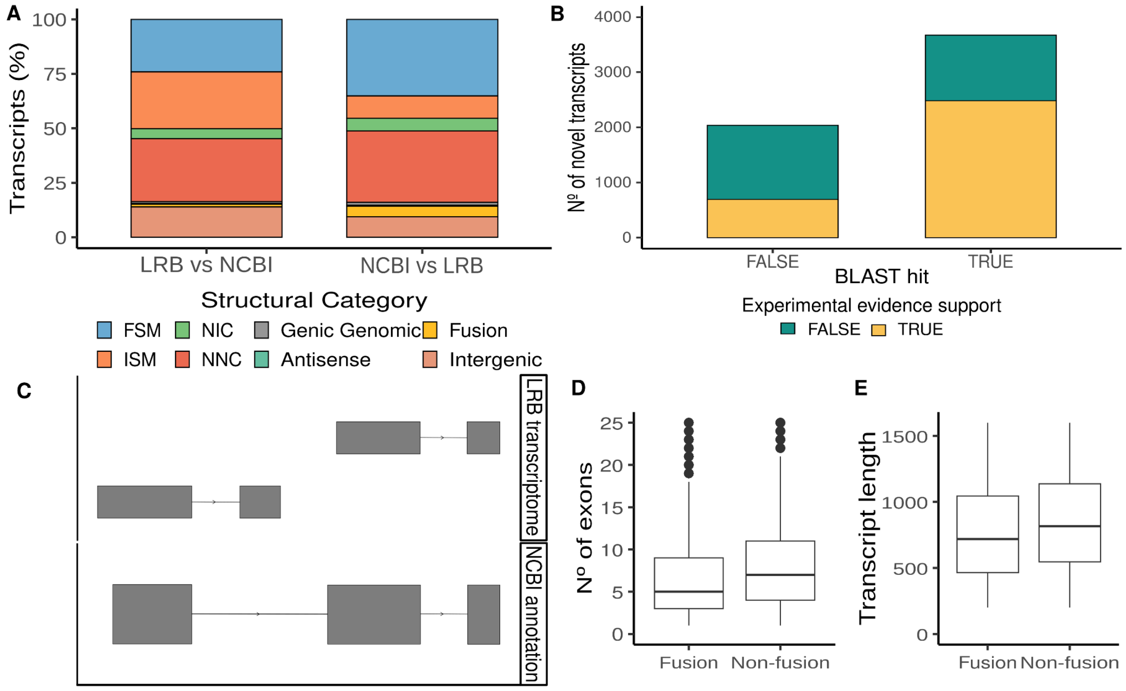

Supplemental Figure S6. Comparison of the Long-read-based (LRB) annotation of the Florida Manatee with the short-read-based NCBI annotation. (A) SQANTI3 isoform classification of the LRB annotation vs. the NCBI annotation (left) and isoform classification of the NCBI annotation vs. the LRB annotation (right). (B) Number of new transcripts classified by BLAST hit presence. The first bar represents transcripts without a BLAST hit, while the second bar represents transcripts with a BLAST hit. Each bar is further divided into sections by color, indicating the proportion of transcripts with experimental support. This differentiation highlights the comparative prevalence of experimental validation among newly identified transcripts with BLAST hit. (C) Comparison between NCBI annotation for the adenylate kinase isoenzyme 1-like and the associated transcripts for this fusion isoform present in the LRB annotation. Comparison between the number of exons (D) and transcript length (E) of LRB supported genes associated with fusion isoforms and non-fusion isoforms in the NCBI annotation.

A

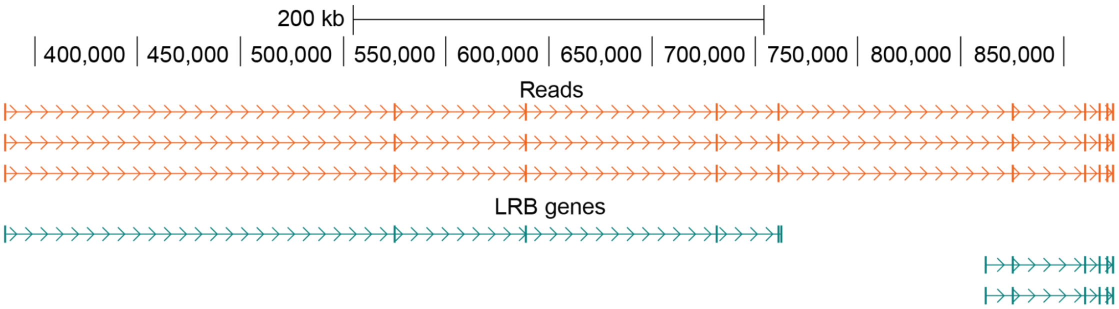

B

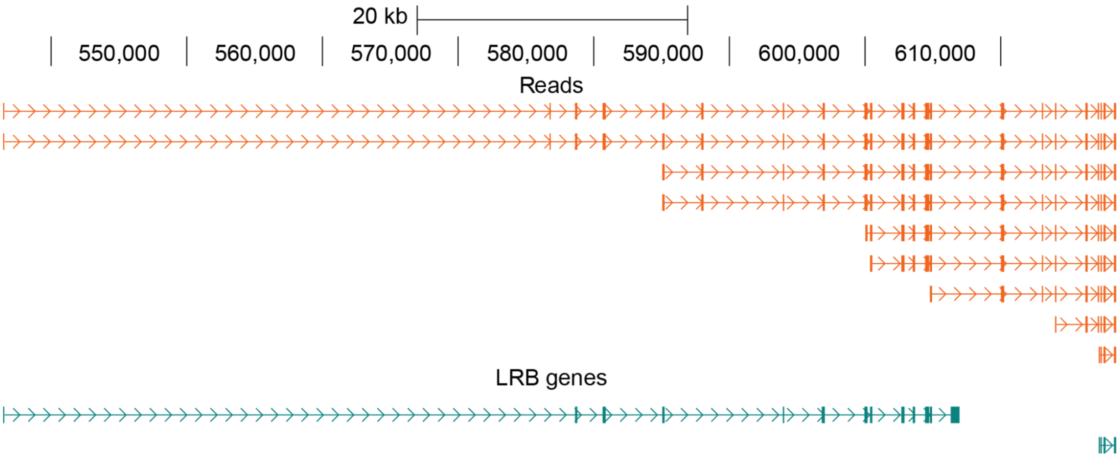

Supplemental Figure S7. Characterization of fragmented genes. (A) Example of a *locus* where the gene (green) is fragmented without long-read (orange) support for any of the fragments. (B) An example of a *locus* is where one of the fragments in LRB annotation has long-read support.

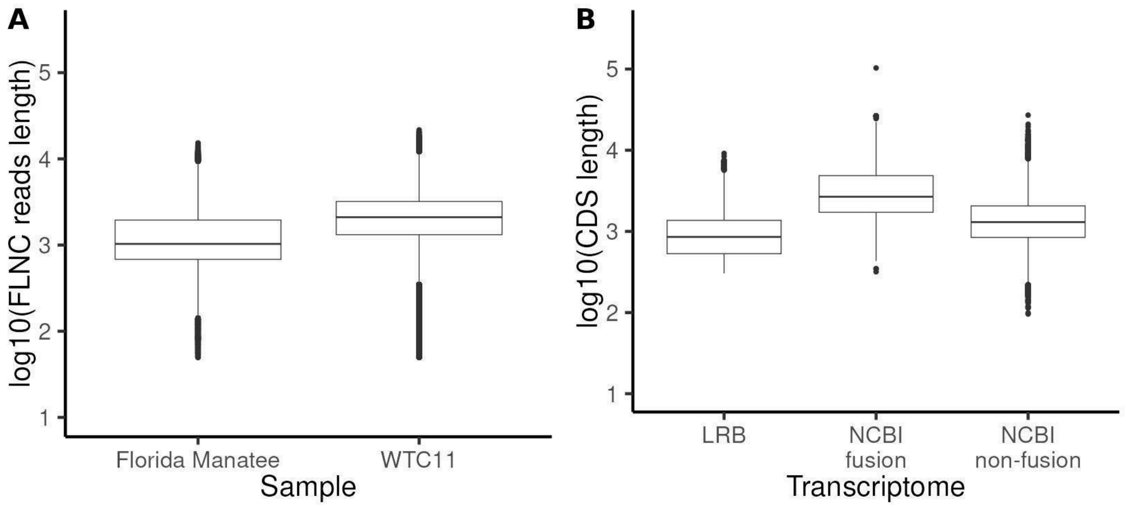

Supplemental Figure S8. Distribution of reads and CDSs lengths. (A) The FLNC PacBio reads for the Florida manatee were, on average, shorter than the FLNC reads obtained from the WTC11 cell line. (B) The long-read-based annotation (LRB) for the Florida manatee exhibited shorter CDSs than the NCBI annotation, particularly for genes in NCBI that overlapped multiple genes in the LRB annotation (NCBI fusion genes).
